# Rush Medical College

**Published:** 1846-12

**Authors:** 


### PART IV.—EDITORIALS.

### ARTICLE XII.

### RUSH MEDICAL COLLEGE.

The lectures in this institution commenced, as usual, on the first Monday of November, to a much larger class than has been in attendance at any former session.

As Chicago is taking rank amongst the large cities of the west, her medical school seems to be rapidily growing in the favor and confidence of the medical public. And we see no good reason why it shall not continue to increase in the facilities for instruction, with the rapid growth of the city, and at once take a stand amongst the first institutions of the kind. It is a well established fact, that no school of medicine can give great facilities for the study of anatomy, or for clinical instruction, unless located in a populous city. And without these, it is scarcely worth while for the student to spend his time and money, in attending upon its teachings; as the former is the foundation, and the latter the capstone of a medical education, which, without them, is like an edifice without anything to stand upon, or to cover it,—an early and a positive ruin.

In these respects, Chicago, which contains about fifteen thousand inhabitants, offers facilities equal to any city of its size in the country, which will increase, to correspond with any probable increase in the classes of the college.

The city dispensary (the foundation of a hospital to be established) now affords an extensive field for clinical observation, and the instructions in it are amongst the most valuable of the course.

The college library, to be used for reference, by an arrangement of the faculty, will regularly increase year after year, and now furnishes most of the works to which a teacher or student could desire to refer; in addition to which, are regularly received all of the medical periodicals published in the United States.

The museum is rapidly filling up, and in a few years will be one of the best in the western country.

The friends of the institution are earnestly requested to preserve, and send in, all valuable specimens in morbid anatomy, botany, &c., they may find, which shall be labeled with their names and carefully preserved.

The members of the faculty, are required to remain in attendance, during the whole term of the session, and the course will be as full and complete as in any school in the country.

This arrangement is the more gratifying since a number of small schools, in the country, are in the habit of giving in a few weeks, sometimes not over three or four, their full course on several of the branches.

If we understand rightly what is necessary to constitute a full course of instruction, as customary in all medical schools of good standing, (though entirely too brief even then,) it is to have a lecture of one hour each day of the session upon chemistry, practice, anatomy, and surgery; unless the two latter are associated and taught by the same professor, as they formerly were in most schools, when but nine lectures per week were given upon the two branches; four lectures per week upon each of the branches of materia medica, and obstetrics, &c., with the regular anatomical demonstrations. And we hold that no school giving less instruction than this, should be recognized by other colleges as giving a full course of lectures.

With the advantages of a location in a city, that must soon become the principal mart, for a large portion of three or four adjoining states and territories, a full and thorough course of instruction in its several departments, a well selected library, a museum containing a large number of specimens in morbid anatomy, special and surgical anatomy, mineralogy, geology, botany, and a complete cabinet in materia medica, a good set of chemical apparatus, and the preparations, machines, and instruments, necessary for fully demonstrating the courses on surgery and obstetrics; a full supply of the material for demonstrating anatomy, its extensive clinical instructions in the hospital, and the great liberality of the city towards it, the Rush Medical College must soon be one of the very best institutions of the kind in the country. E.

